# Diagnostic outcomes of bone marrow aspirate and trephine biopsies performed at a hospital in KwaZulu-Natal, South Africa

**DOI:** 10.4102/ajlm.v9i1.1028

**Published:** 2020-02-25

**Authors:** Wanda S. Tshabalala, Somasundram Pillay, Douglas P.K. Wilson

**Affiliations:** 1 Department of Internal Medicine, Grey’s Hospital, University of KwaZulu-Natal, Pietermaritzburg, South Africa; 2 Department of Internal Medicine, Edendale Hospital, Pietermaritzburg complex, University of KwaZulu-Natal, Pietermaritzburg, South Africa

**Keywords:** age, human immune virus, indications, outcomes, haematological malignant diseases, diffuse large B cell lymphoma, bone marrow aspirate and trephine biopsy

## Abstract

**Background:**

Bone marrow aspiration and trephine biopsy (BMAT) are widely performed in adults to evaluate haematological and malignant conditions. However, the diagnostic yield from the procedure in unselected patients in the South African public sector has not previously been described.

**Objectives:**

We identified the main indications and most common diagnoses encountered for BMAT and described the demographic and blood profiles of patients, including HIV-positive patients, who had undergone the procedure at a tertiary hospital in KwaZulu-Natal.

**Methods:**

We retrospectively reviewed laboratory data from January 2016 to December 2016 for all patients aged ≥ 13 years who underwent the procedure and stratified findings by demographic data.

**Results:**

Among 120 BMAT biopsies studied, 80 (67%) cases were performed to evaluate suspected malignancy and a further 40 (33%) cases for non-malignant indications. The main indications for bone marrow examination were: cytopenias 38 (32%), lymphoma 35 (29%), leukaemia 21 (18%), and multiple myeloma 17 (14%). BMAT results revealed that 60 cases (50%) were malignant in origin, 30 cases (25%) were non-malignant and 30 cases (25%) were classified as normal. The common diagnoses were: leukaemia, 24 (20%); multiple myeloma, 16 (13%) and lymphoma, 13 (11%). Cases aged ≥ 50 years were more likely to have a malignant diagnosis (odds ratio: 5.8 (95% confidence interval: 2.2–17.1) p-value < 0.001).

**Conclusion:**

The diagnostic yield of BMAT was high, with significant abnormalities detected in three quarters of cases. Haematological malignancy was the more common diagnosis. Increasing age was associated with an increase in reporting of haematology malignancy.

## Introduction

Bone marrow examination may provide vital information in the diagnosis and monitoring of haematological disease.^1^ Bone marrow examination can be very effective when it is utilised in conjunction with a good clinical assessment. In some cases, diagnosis cannot be reached solely on an assessment of peripheral blood samples alone, and in these situations examination of the bone marrow is crucial.^2^

Several South African studies have evaluated outcomes from the procedure in selected cohorts with HIV, tuberculosis or lymphoma. Karsteadt et al., in their study examining the benefits of bone marrow examination in HIV-positive adults, showed that a low CD4 count (< 100 cells/*μ*L) had a higher diagnostic yield in granulomatous disease of the bone marrow.^3^ In 2014, Naidoo et al. studied the outcome of histological comparison to cytology in antiretroviral-therapy-naïve vs. antiretroviral-therapy-experienced HIV-positive patients presenting with peripheral blood cytopenias and found higher rates of granulomatous disease and pure red cell aplasia in antiretroviral-therapy-naïve patients.^4^ More recently in 2018, in a study conducted by Philips and Opie to assess the use of bone marrow examination in lymphoma sufferers in South Africa, it was shown that out of the 1215 Bone marrow aspiration and trephine (BMAT) records, bone marrow involvement was most commonly present in non-Hodgkin’s lymphoma (43.7%), followed by high-grade B subtypes (28.9%) and Hodgkin’s lymphoma (35.7%).^5^

More than 200 bone marrow examinations are performed annually by our institution. A bone marrow examination is a crucial test that comes at a cost to the health institution. In the interest of healthcare cost effectiveness measures, we avoid performing BMAT for treatable nutritional disorders with peripheral blood cytopenias. Serum tests to confirm and assess for nutritional deficiencies are freely available at our institution. Patients with cytopenias are therefore routinely screened for nutritional deficiencies such as vitamin B12 and folate deficiency, which can mimic leukaemia and had been reported in literature case studies.^6^

Studies have demonstrated that the most common reasons for bone marrow examination were for diagnoses of haematological malignancies.^7,8^ The spectrum of haematological disease at our institution has not been previously studied and reported. We therefore undertook a retrospective analysis of sequential BMAT biopsies performed over a 12-month period to evaluate the indications for and diagnostic yield from the procedure.

## Methods

### Ethical considerations

Ethical clearance was obtained from the Biomedical Research Ethics Committee, University of KwaZulu-Natal (Ethical Clearance number: BE465/17).

### Study site and design

The haematology unit at Grey’s Hospital, Pietermaritzburg, South Africa, offers tertiary inpatient and outpatient services to the western half of KwaZulu-Natal and performs all the adult BMAT procedures for the institution. We performed a retrospective cross-sectional study on results from BMAT performed at Grey’s Hospital on individuals older than age 13 years from 01 January 2016 to 31 December 2016.

### Data collection

The following clinical, demographic, and biochemical parameters were extracted from the BMAT records: age; sex; HIV status; cluster of differentiation 4 (CD4) lymphocyte count cells/*µ*L; indication for BMAT; final diagnostic outcome of BMAT; BMAT quality and trephine size; white cell count cells × 10^9^/L; haemoglobin grams/dL; platelet count cells × 10^9^.

### Bone marrow and trephine sampling and definition

We retrospectively analysed results from BMAT performed by physicians at our institution’s clinical haematology unit during routine patient care. The National Health Laboratory Service processed these biopsy specimens in accordance with the recommendations of the World Health Organization.^9^ The World Health Organization recommends a minimum of 1.5 cm length for a size of trephine core biopsy as adequate assessment.^9^ BMAT results were included if: (1) Patient was older than age 13 years at the time of the biopsy; and (2) BMAT was reported to have adequate aspirate or trephine quality to reach final diagnoses.

### Statistical analysis

Analyses were performed using Analyse-it Ultimate Edition (Analyse-it Ltd, Leeds, United Kingdom). Distributions of continuous data were determined using the Shapiro Wilk test and odds ratios with 95% confidence intervals for categorical data calculated using the Fisher exact test.

## Results

A total of 204 cases were received from the National Health Laboratory Service. One hundred and twenty cases met the inclusion criteria. Eighty-four cases that did not meet the inclusion criteria included 68 cases excluded for being below the cut-off age of 13 years; 15 cases were excluded because they were out of the specified time range; and one case that had a poor trephine biopsy quality. Seven of the 120 BMAT cases had a poor bone marrow aspirate but had a good quality bone marrow trephine biopsy. These cases were treated as eligible, as a diagnosis could still be made on the information provided by the trephine biopsy.

### Demographics

**FIGURE 1:**
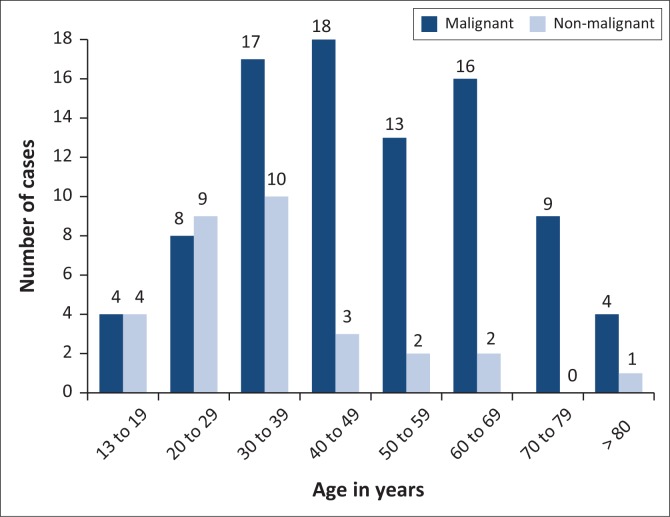
Distribution of malignant and non-malignant conditions according to age groups of 120 bone marrow aspirates and trephine biopsies performed at a hospital in KwaZulu-Natal, South Africa, January 2016 to December 2016.

Malignant diagnoses were predominantly reported in all age groups older than age 30 years (Figure 1). The study consisted of 63 (52.5%) male cases and 57 (47.5%) female cases with a median age of 40 years (interquartile range [IQR]: 30–60 years). Age ≥ 50 years was associated with an increase in reporting of malignancy (odds ratio: 5.8; 95% confidence interval: 2.2–17.1; *p* < 0.001).

### Indications for performing a bone marrow aspirate and trephine biopsy

The indications for BMAT performed at our institution included 38 cases with cytopenias (32%), 35 with lymphoma (29%), 21 with leukaemia (18%), and 17 with multiple myeloma (14%) (Table 1).

**TABLE 1:** Indications for performing bone marrow aspirate and trephine biopsies on an unselected group of 120 patients at a hospital in KwaZulu-Natal, South Africa, January 2016 to December 2016.

Indications	*n*	%
**Malignant indications**		
**Lymphoma**		
Non-Hodgkin’s		
Diffuse large B cell	27	22.5
Mantle cell lymphoma	1	0.8
Burkett’s lymphoma	1	0.8
Hodgkin’s lymphoma	6	5.0
**Leukaemia**		
Chronic lymphocytic	9	7.5
Chronic myeloid leukaemia	5	4.2
Acute leukaemia non-specific	5	4.2
Acute myeloid	2	1.7
**Plasma cell dyscrasias**		
Multiple myeloma	17	14.2
**Myeloproliferative neoplasms**		
Polycythaemia vera	2	1.7
Essential thrombocytosis	2	1.7
Breast cancer	1	0.8
Lung cancer	1	0.8
Myelodysplastic syndrome	1	0.8
**Total number of malignant indications**	**80**	**67.0**
**Non-malignant indications**		
**Cytopenias**		
Pancytopenia	19	15.8
Anaemia	8	6.7
Thrombocytopenia	7	5.8
Bicytopenia	3	2.5
Neutropenia	1	0.8
Eosinophilia	1	0.8
Hereditary spherocytosis	1	0.8
**Total number of non-malignant indications**	**40**	**33.0**

### Indication of bone marrow aspirate and trephine biopsy among HIV-positive cases

HIV-positive cases accounted for 46 (38%) of the 120 BMATs reported with a median CD4 lymphocyte count of 241 cells/*µ*L (IQR: 178.7–303.7). Peripheral blood cytopenias and lymphomas were the common indications for bone marrow examination in the HIV-positive cohort (Table 2). A diagnosis of diffuse large B cell lymphoma (DLBCL) made on a peripheral lymph node biopsy was an indication for BMAT in 22 (47.8%) of 46 cases known to be HIV-positive.

**TABLE 2:** Indications for bone marrow examination of 46† cases reported to be HIV-positive at a hospital in KwaZulu-Natal, South Africa, January 2016 to December 2016.

Indications of BMAT	*n*	%
Diffuse large B cell lymphoma	22	47.8
Pancytopenia	10	21.7
Anaemia	7	15.2
Bicytopenia	3	6.5
Hodgkin’s lymphoma	2	4.3
Thrombocytopenia	1	2.2
Chronic lymphocytic leukaemia	1	2.2

BMAT, Bone marrow aspirate and trephine biopsy.

† , Positive in a study of 120 BMAT.

Eighty-one percent (22/27) of the overall DLBCL cases were HIV-positive. Less than 1% of the cases with DLBCL had bone marrow infiltration. Sixty percent of HIV-positive cases with a diagnosis of DLBCL had a CD4 count ≥ 200 cells/*µ*L. The median full blood count indices among HIV-positive cases were: white blood cell count, 6.43 × 10^9^/L (IQR: 5.030–8.10); haemoglobin, 7.9 (IQR: 6.8–9.00) g/dL; and platelet count, 243.0 (IQR: 191.0–365.0) × 10^9^/L.

### Diagnostic outcomes of the bone marrow examination

Reported diagnoses for this study revealed 60 cases (50%) with malignancy, 30 cases (25%) were reported as non-malignant and a further 30 cases (25%) revealed a normal bone marrow (Table 3). The most common diagnoses were (in descending order of frequency): 24 cases of leukaemia (20%); 16 of multiple myeloma (13%) and 13 of lymphoma (11%). Acute leukaemia accounted for 46% (11/24 cases) of all diagnosed leukaemia cases when compared to chronic leukaemia. The median full blood count indices for leukaemia cases were: white cell count 93.00 × 10^9^/L (IQR: 36.03–159.45); haemoglobin 7.3 g/dL (IQR: 7.00–11.30); and platelet count 130 × 10^9^/L (IQR: 82.0–241.0). There were five cases reported to have granulomas, one case with a non-specific granuloma and a further four cases with tuberculosis granulomas. Three of the cases diagnosed with tuberculosis granulomas were HIV-positive; all had CD4 counts less than 200 cells/*µ*L.

**TABLE 3:** Reported final diagnoses from bone marrow aspirate and trephine results of an unselected group of 120 patients at a hospital in KwaZulu-Natal, South Africa, January 2016 to December 2016.

Outcomes	*n*	%
**Malignant outcomes**		
**Lymphomas**		
Hodgkin’s lymphoma	9	7.5
Diffuse large B cell lymphoma	3	2.5
Mantle cell lymphoma	1	0.8
**Plasmadyscrasias**		
Multiple myeloma	16	13.3
**Chronic Leukaemia**	-	-
Chronic lymphocytic	9	7.5
Chronic myeloid	4	3.3
**Acute Leukaemia**		
Acute myeloid	5	4.2
Acute lymphocytic	3	2.5
Acute leukaemia non-specific	2	1.7
Acute promyelocytic	1	0.8
**Myeloproliferative neoplasms**		
Polycythaemia vera	3	2.5
Essential thrombocytosis	1	0.8
Breast cancer	1	0.8
Lung cancer	1	0.8
Myelodysplastic syndrome	1	0.8
**Total number of malignant outcomes**	**60**	**50.0**
**Non-malignant outcomes**		
**Cytopenias**		
Pancytopenia due to peripheral destruction	7	5.8
Aplastic anaemia	5	4.2
Thrombocytopenia due to peripheral destruction	5	4.2
Pure red cell aplasia	4	3.3
Megaloblastic anaemia	1	0.8
Neutropenia due to sepsis	1	0.8
**Granulomas**		
Tuberculosis	4	3.3
Non-specific granuloma	1	0.8
Eosinophilia	1	0.8
Hereditary spherocytosis	1	0.8
**Total number of non-malignant outcomes**	**30**	**25.0**

**Total number of normal bone marrow results**	**30**	**25.0**

## Discussion

Bone marrow aspirate and trephine biopsies in our institution were performed for a wide array of haematological conditions ranging from highly aggressive to non-benign malignancies. Our findings were similar to some international studies conducted in Nigeria, Nepal, Saudi Arabia, Iran, India, and Ghana, all of which had reported haematological malignant disease as a common indication and outcome for a bone marrow examination.^7,8,10,11,12,13^ In our study there were 30 normal bone marrow results with the majority of these cases being BMAT performed as part of the staging process for treatment of malignancies.

Since South Africa is a country known to have a high prevalence of HIV infection, the use of BMAT in the diagnosis of tuberculosis is not standard practise at our institution. We have access to diagnostic tools such as Gene Xpert and tuberculosis culture, which can be used in testing various body fluids of individuals with suspected tuberculosis infection, as well as urine lipoarabinomannan, which can assist with early diagnosis of disseminated tuberculosis. Therefore, many patients with tuberculosis would have been diagnosed before BMAT was considered by the attending clinicians. For this reason our study reported low rates (4/120) of tuberculosis diagnosis on BMAT when compared to other South African studies that demonstrated higher tuberculosis diagnoses on BMAT samples.^4,14^ These studies included: a study that looked at 410 case samples of adult and paediatric BMAT submitted for excluding tuberculosis, which showed a 32.5% tuberculosis prevalence, and a study that assessed bone marrow morphology in 74 patients, in which comparing morphology between patients on antiretroviral therapy and patients naïve to antiretroviral therapy showed a 31% tuberculosis prevalance.^4,14^ Both of these studies had higher tuberculosis prevalence due to their study populations being cases with high risk for tuberculosis.

Workup for cases presenting with cytopenias formed a great majority of BMAT cases in our study. These results are similar to studies done to assess the use of BMAT in hospitals in Pakistan and India.^15,16^ Cytopenias are well documented, multifactorial complications of HIV infection.^4,5^ Our study demonstrated that 46% of HIV-positive participants had one or more forms of cytopenia: anaemia, pancytopenia, bicytopenia and thrombocytopenia. A study conducted in Uganda demonstrated that 65% of antiretroviral-therapy-naïve HIV-positive participants had a form of cytopenia.^17^ We could not draw any conclusion with this result, as we had not assessed the status of antiretroviral therapy use by participants.

Age is known to be one of the important risk factors for developing haematological malignancy. In our study increasing age was associated with an increased prevalence of malignancy. The age group older than age 70 years had the highest frequency of malignancy when compared to the younger age study population groups for this study. This outcome is similar to other global studies that have assessed increasing age as a risk factor for developing malignancy.^18,19,20^ A study of lymphoid neoplasms in the United States demonstrated that the incidence of haematological malignancy in cases older than age 75 years increased by approximately 1.4% for DLBCL and 1.8% for follicular lymphoma per year.^19^

DLBCL is associated with high rates of HIV infection among cases.^21^ Our HIV and DLBCL burdens are in keeping with the current rates in most countries throughout the world.^4,21^ HIV infection was highly prevalent at 81% among the cases that had undergone bone marrow examination for the staging of the DLBCL. This finding about prevalence of HIV infection in cases with DLBCL was similar to results of a study done at a South African hospital in 2016 which reported the prevalence of HIV as 81% in 139 patients with DLBCL.^22^ Sixty percent of cases of DLBCL in our study had a CD4 lymphocyte count ≥ 200 cell/*µ*L. The median CD4 count for our DLBCL group was 259 (IQR: 131.2–351.6) cells/*µ*L. This outcome demonstrates that HIV infection placed patients at a higher risk for developing this malignancy and occurrence of the malignancy did not depend on low CD4 count. In the United States, a report on 41 patients diagnosed with DLBCL concluded that the blood CD4 level was a lesser factor in predicting the occurrence of DLBCL.^23^ The median CD4 count of their study was 400 cells/*µ*L. It has been reported that a haemoglobin value of less than 10 g/dL and white cell count less than 4 × 10^9^ significantly increases the likelihood of bone marrow involvement in non-Hodgkin’s lymphoma.^24^ About 99% of cases reported for DLBCL in our study had no bone marrow infiltration. A large percentage (72%) of our cases without an infiltration had anaemia with a mean haemoglobin of 9.92 g/dL, a mean normal white cell count 9.91 × 10^9^/L and mean platelet counts of 340 × 10^9^/L. Other reasons for the development of anaemia, such as: myelosupression secondary to chemotherapy, anaemia associated with chronic disease, autoimmune haemolytic anaemia, iron deficiency and tumour infiltration of bone marrow, need to be explored in patients with DLBCL to identify a potential aetiology.^25^ The majority of the BMATs of cases with DLBCL were done as part of treatment planning. Our hospital uses computerized tomography (CT) scanning and bone marrow examination for staging malignancy. Our access to flourine-18-flourodeoxyglucose position emission tomography (PET) scanning is limited in our setting. PET scanning has been recommended in the literature as a superior staging modality for bone marrow involvement in lymphoma sufferers.^26^ Medical literature has documented the limitations of bone marrow examination in patients with aggressive DLBCL and early lymphoma.^26^ Other studies suggest that PET/CT scanning is more useful in assessing the involvement of the bone marrow.^26,27^ Improved access to PET/CT scanning would reduce the frequencies of bone marrow examinations in lymphoma sufferers, as most studies suggest that it should be considered as a first-line examination of bone marrow in lymphoma patients.^26,27^ Bone marrow infiltration in DLBCL can easily be missed as most DLBCL infiltration are focal rather than diffuse.^26^ PET/CT has a much more superior sensitivity (94% vs. 24%) and a higher negative predictive value (98% vs. 80%) when compared to a bone marrow examination.^27^

In our study there was only one case reported as nutritional deficiency and that was megaloblastic anaemia due to folate deficiency. Our findings are in line with the current practice in South Africa, which states that bone marrow examination is done to prove another diagnosis other than confirming nutritional deficiencies. In our current limited resource situation, the practice is to correct reversible factors such as nutritional disorders having a haematological impact first before subjecting patients to more expensive and invasive investigations. In the South African health system, both in the private and public sector, there is easy access to testing for nutritional deficiencies without having first to do BMAT. This is possibly the reason why there was a paucity of BMAT done in our study that showed nutritional deficiencies. We expect this trend to continue in our setting because of the required screening of nutritional deficiencies in our South African institutions.

### Limitations

As this was a retrospective study, we were not able to follow up on the end-point of the cases that had DLBCL or other malignancies nor were we able to assess the number of patients that were on antiretroviral medications. Since the study site is a referral centre, the patients that are referred across come mostly with initial exclusion of a reversible cause for bone marrow failure. Our results might also be influenced by the study population as it only represents patients who were treated by the haematologists with experience in such cases.

### Recommendations

Based on the odds ratio of cases older than age 50 years in this study, we recommend treating physicians to have a low threshold for performing BMAT in patients aged 50 years and older who present with any cytopenias, as this has shown an increased likelihood of a malignant diagnosis. We also recommend an early peripheral tissue biopsy in conjunction with BMAT in HIV-positive individuals presenting with constitutional symptoms of malignancy, lymphadenopathy, or cytopenias for early identification of life-threatening malignancies.

### Conclusion

Haematological malignant disease was the most common indication and outcome reported for bone marrow examination. The majority of BMAT done for the staging of DLBCL did not reveal evidence of bone marrow infiltration. Increasing age was found to be associated with reporting of haematological malignancies. Nutritional disorders were rarely reported as an indication for BMAT.

## References

[1] Burkhardt R, Frisch B, Bartl R Bone marrow biopsy in haematological disorders. J Clin Pathol. 1982;35(3):257–284. 7040489 10.1136/jcp.35.3.257 PMC497529

[2] Thelm H, Diem H, Haferlach T Colour atlas of hematology practical microscopic and clinical diagnosis. 2nd ed. Stuttgart: Thieme Verlag, 2004; p. 20–27.

[3] Karstaedt AS, Pantanowitz L, Omar T, Sonnendecker HE, Patel M The utility of bone-marrow examination in HIV-infected adults in South Africa. QJM. 2001;94(2):101–105. 10.1093/qjmed/94.2.101 11181986

[4] Naidoo S, Naicker VL Retrospective comparison of cytological and histological bone marrow morphology in adult antiretroviral-naïve and antiretroviral experienced human immunodeficiency virus-infected patients with peripheral blood cytopaenias. S Afr J Infect Dis. 2016;31(2):50–56. 10.1080/23120053.2016.1128147

[5] Phillips L, Opie J The utility of bone marrow sampling in the diagnosis and staging of lymphoma in South Africa. Int J Lab Hematol. 2018;40(3):276–283. 10.1111/ijlh.12782 29427399

[6] Cacoub P, Gatfosse M, Derbel A, Chapelon C, Verny C, Godeau P Vitamin deficiency-induced pancytopenia mimicking leukemia. Three cases. Presse Med. 1991;20(33):1603–1606. 1835075

[7] Dapus DO, Damen JG Diagnostic outcome of bone marrow aspiration in a new center in Nigeria. Glob Adv Res J Med Med Sci. 2012;1(7):166–177.

[8] Jha A, Suyami G, Adhikari RC, Panta AD, Jha R Bone marrow examination in cases of pancytopenia. J Nepal Med Assoc. 2008;47(169):12–17. 18552886

[9] Swerdlow SH, Campo E, Harris NL, et al WHO classification of tumours of haematopoietic and lymphoid tissues. Lyon: IARC; 2008.

[10] Bashawri LA Bone marrow examination indications and diagnostic values. Saudi Med J. 2002;23(2):191–196. 11938397

[11] Mirzai AZ, Hosseini N, Sadeghipour A Indications and diagnostic utility of bone marrow examination in different bone marrow disorders in Iran. Lab Hematol. 2009;15(4):38–44. 10.1532/LH96.09009 19923104

[12] Shastry SM, Kolte SS Spectrum of hematological disorders observed in one-hundred and ten consecutive bone marrow aspirations and biopsies. Med J Dr. D.Y. Patil Uni. 2012;5(2):118–121. 10.4103/0975-2870.103334

[13] Bedu-Addo G, Ampem Amoako Y, Bates I The role of bone marrow aspirate and trephine samples in haematological diagnoses in patients referred to a teaching hospital in Ghana. Ghana Med J. 2013;47(2):74–78. 23966743 PMC3743116

[14] Sedick Q, Vaughan J, Pheeha T, Alli NA Bone marrow aspirates microscopy v. bone marrow trephine biopsy microscopy for detection of mycobacterium tuberculosis infection. S Afr Med J. 2015;105(9):773–775. 10.7196/SAMJnew.8171 26428979

[15] Ahmad SQ, Khan OU, Zafar N Utility of bone marrow examination in a secondary care hospital. JRMC. 2011;15(1):40–41.

[16] Bashir N, Musharaf B, Reshi R, Jeelani T, Rafiq D, Angmo D Bone marrow profile in hematological disorders: an experience from a tertiary care centre. Int J Adv Med. 2018;5(3):608–613. 10.18203/2349-3933.ijam20182111

[17] Kyeyune R, Saathoff E, Ezeamama A, Löscher T, Fawzi W, Guwatudde D Prevalence and correlates of cytopenias in HIV-infected adults initiating highly active antiretroviral therapy in Uganda. BMC Infect Dis. 2014;14:496 10.1186/1471-2334-14-496 25209550 PMC4165997

[18] Hassan M, Abedi-Valugerdi M Hematologic malignancies in elderly patients. Haematologica. 2014;99(7):1124–1127. https://doi.org/10.3324%2Fhaematol.2014.107557 24986872 10.3324/haematol.2014.107557 PMC4077070

[19] Morton LM, Wang SS, Devesa SS, Hartge P, Weisenburger DD, Linet MS Lymphoma incidence patterns by WHO subtype in the United States. Blood. 2006;107(1):265–276. 10.1182/blood-2005-06-2508 16150940 PMC1895348

[20] Ohshima K, Suzumiya J, Kikuchi M The World Health Organization classification of malignant lymphoma: Incidence and clinical prognosis in HTLV-1-endemic area of Fukuoka. Pathol Int. 2002;52(1):1–12. 10.1046/j.1440-1827.2002.01308.x 11940200

[21] Hicks L, Cheung M, Boro J, Ezzat H, Leitch H HIV-associated diffuse large B cell lymphoma: Determinants of survival in the era of rituximab and HAART. Blood. 2010;116(21):2835 10.1182/blood.V116.21.2835.2835

[22] Machialo JT Diffuse large B cell lymphoma in adults at Chris Baragwanath hospital [homepage on the Internet]. Johannesburg: Witwatersrand University; 2016 [cited 2018 Aug 3]. Available from: http://hdl.handle.net/10539/23175

[23] Al-Saleem TI, Dulaimi E, Millenson MM, Smith MR, Judd J, Li T, Borghaei H Low blood absolute CD4 counts is associated with inferior progression free survival in diffuse large B-cell lymphoma independent of age and IPI. Blood. 2014;124(21):5420 10.1182/blood.V124.21.5420.5420

[24] Lim ST, Tao M, Cheung YB, Rajan S, Mann B Can patients with early-stage diffuse large B-cell lymphoma be treated without bone marrow biopsy? Ann Oncol. 2005;16(2):215–218. 10.1093/annonc/mdi050 15668272

[25] Sung HJ, Kim SJ, Lee JH, et al Persistent anemia in a patient with diffuse large B cell lymphoma: pure red cell aplasia associated with latent Epstein-Barr virus infection in bone marrow. J Korean Med Sci. 2007;22:167–170. 10.3346/jkms.2007.22.S.S167 17923747 PMC2694371

[26] Khan AB, Barrington SF, Mikhaeel NG, et al PET-CT staging of DLBCL accurately identifies and provides new insight into the clinical significance of bone marrow involvement. Blood. 2013;122(1):61–67. 10.1182/blood-2012-12-473389 23660958

[27] Berthet L, Cochet A, Kanoun S, et al In newly diagnosed diffuse large B-cell lymphoma, determination of bone marrow involvement with ^18^F-FDG PET/CT provides better diagnostic performance and prognostic stratification than does biopsy. J Nucl Med. 2013;122(1):61–67. 10.2967/jnumed.112.114710 23674577

